# Excimer based fluorescent pyrene–ferritin conjugate for protein oligomerization studies and imaging in living cells†

**DOI:** 10.1039/c8ra00210j

**Published:** 2018-04-03

**Authors:** Irene Benni, Matilde Cardoso Trabuco, Enrico Di Stasio, Alessandro Arcovito, Alberto Boffi, Francesco Malatesta, Alessandra Bonamore, Simone De Panfilis, Valeria de Turris, Paola Baiocco

**Affiliations:** Department of Biochemical Sciences “Alessandro Rossi Fanelli”, Sapienza University of Rome P.le A. Moro 5 00185 Rome Italy; Molirom srl *via* Ravenna 8 00161 Rome Italy; Institute of Biochemistry and Clinical Biochemistry, Catholic University Largo Francesco Vito, 1 00168 Rome Italy; Institute of Molecular Biology and Pathology, National Research Council P.le A. Moro 7 00185 Rome Italy; Center for Life Nano Science@Sapienza, Istituto Italiano di Tecnologia V.le Regina Elena 291 00161 Rome Italy paola.baiocco@iit.it

## Abstract

Ferritin self-assembly has been widely exploited for the synthesis of a variety of nanoparticles for drug-delivery and diagnostic applications. However, despite the crucial role of ferritin self-assembly mechanism for probes encapsulation, little is known about the principles behind the oligomerization mechanism. In the present work, the novel “humanized” chimeric Archaeal ferritin HumAfFt, displaying the transferrin receptor-1 (TfR1) recognition motif typical of human H homopolymer and the unique salt-triggered oligomerization properties of *Archaeoglobus fulgidus* ferritin (AfFt), was site-selectively labeled with *N*-(1-pyrenyl)maleimide on a topologically selected cysteine residue inside the protein cavity, next to the dimer interface. Pyrene characteristic fluorescence features were exploited to investigate the transition from a dimeric to a cage-like 24-meric state and to visualize the protein *in vitro* by two photon fluorescence microscopy. Indeed, pyrene fluorescence changes upon ferritin self-assembly allowed to establish, for the first time, the kinetic and thermodynamic details of the archaeal ferritins oligomerization mechanism. In particular, the magnesium induced oligomerization proved to be faster than the monovalent cation-triggered process, highly cooperative, complete at low MgCl_2_ concentrations, and reversed by treatment with EDTA. Moreover, pyrene intense excimer fluorescence was successfully visualized *in vitro* by two photon fluorescence microscopy as pyrene-labeled HumAfFt was actively uptaken into HeLa cells by human transferrin receptor TfR1 recognition, thus representing a unique nano-device building block for two photon fluorescence cell imaging.

## Abstract 2

Pyrene fluorescence changes upon ferritin self-assembly allowed to establish the kinetic and thermodynamic details of the archaeal ferritins oligomerization mechanism and was successfully visualized *in vitro* by two photon fluorescence microscopy.

## Graphical abstract

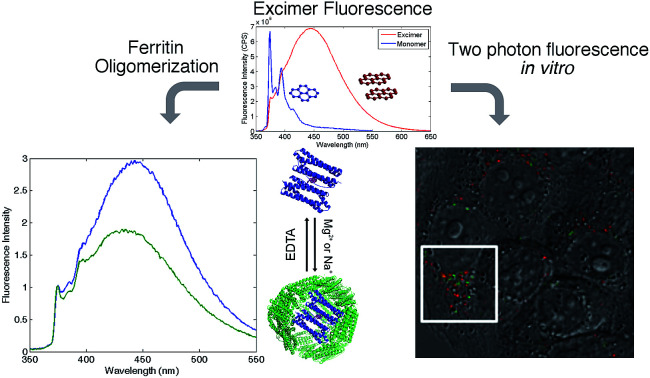

## 1. Introduction

Ferritins are iron storage and transport proteins, found in most living organisms, characterized by a typical tetraeicosameric subunits assembly.^1^ Ferritin assembly results in the formation of a 8 nm diameter central cavity, which can be efficiently loaded with transition metals, drugs, fluorescent molecules or contrast agents.^2–4^ Thus, encapsulated molecules can be delivered through the ferritin nano-cages, to target the transferrin receptor (TfR1, or CD71), highly expressed in actively replicating cells such as tumour cells.^5–7^ These attributes make ferritins and their derivatives a powerful delivery system with potential application in nanomedicine.

Recombinant mammalian ferritins, however, are difficult to disassemble into subunits as they can be dissociated only under extreme conditions, thus rendering the encapsulation of many substrates impractical. Commonly employed cargo encapsulation techniques^8^ involve the disassembly and re-assembly of ferritins cage by pH jump, reaching drastic pH values (pH 2)^9^ which cause a partial and incomplete re-assembly.^10^ On the other hand, the oligomerization mechanism of a unique ferritin from *Archaeoglobus fulgidus* is easily controlled by altering cations concentration in physiological conditions.^11^ In this framework, the humanized ferritin from *Archaeoglobus fulgidus* (HumAfFt)^12^ represents a uniquely suitable scaffold for incorporating diverse substructures at neutral pH while being recognized and internalized in mammalian cells by human transferrin receptor TfR1 as human ferritin H homopolymer.

Surprisingly, however, despite structural determinants of the unique assembly of *Archaeoglobus* ferritin were thorough described^11^ and several studies tried to shed light on the mechanism that drives the monomers association into a mammalian 24-meric ferritin shell^13,14^ under acidic conditions, the unique assembly–disassembly properties of archaeal ferritins were only investigated by light scattering or size exclusion chromatography while the kinetic behavior of the salt-triggered oligomerization is still unknown. Thus, in the present paper, we engineered the HumAfFt ferritin to site-selectively introduce two overlapping pyrene moieties at the ferritin dimer interface with the aim of exploring archaeal ferritins association/dissociation mechanism by exploiting pyrene sensitive fluorescence emission. Pyrene fluorescence properties were widely used for many biological and bioimaging investigations^15–22^ as the probe's high extinction coefficient allows studies of proteins in solution at physiologically relevant concentrations and its high stability and long fluorescent lifetime give it resistance to photodamage and photobleaching.^23^ Pyrene versatility is due to a spatially sensitive fluorescence emission that displays an ensemble of monomer emission peaks and an easily distinguishable red shifted broad peak corresponding to the excited state dimer called excimer.^24,25^ Excimer formation arises when two pyrene molecules, one in the ground state and the other one in the excited state, are located in close proximity (∼10 Å) and are involved in a non-covalent π–π stacking interaction.^26^ Any subtle structural change in a pyrene-labeled protein could alter orientation and distance between the two pyrene molecules involved in the excimer, thus strongly affecting stacking interactions and therefore resulting in a clear shift in the fluorescence emission.^15,24^

Pyrene–HumAfFt bioconjugate, thanks to the peculiar pyrene excimer properties, helped unravelling the kinetic details of the archaeal ferritins oligomerization process, by means of an easily accessible spectroscopic technique and, at the same time, allowed the visualization by two photon fluorescence microscopy of HumAfFt uptake by HeLa cells.

The thermodynamic and kinetic analysis of the assembly process highlighted a complete reversibility and a surprising difference between the association rates induced by mono and divalent cations. In addition, the HumAfFt functionalities were not altered by pyrene-labeling and the bioconjugated ferritin was successfully visualized *in vitro* by using two-photon excimer-based fluorescence microscopy, highlighting a cellular distribution typical of a clathrin-coated endocytosis pathway,^27^ in agreement with the intracellular pattern of the unlabeled HumAfFt previously reported, but with the major advantage of visualizing the protein by low energy wavelength irradiation, thus ensuring longer penetration depth while reducing photodamage.^28^

## 2. Results and discussion

### 2.1 Ferritin design

**Fig. 1:**
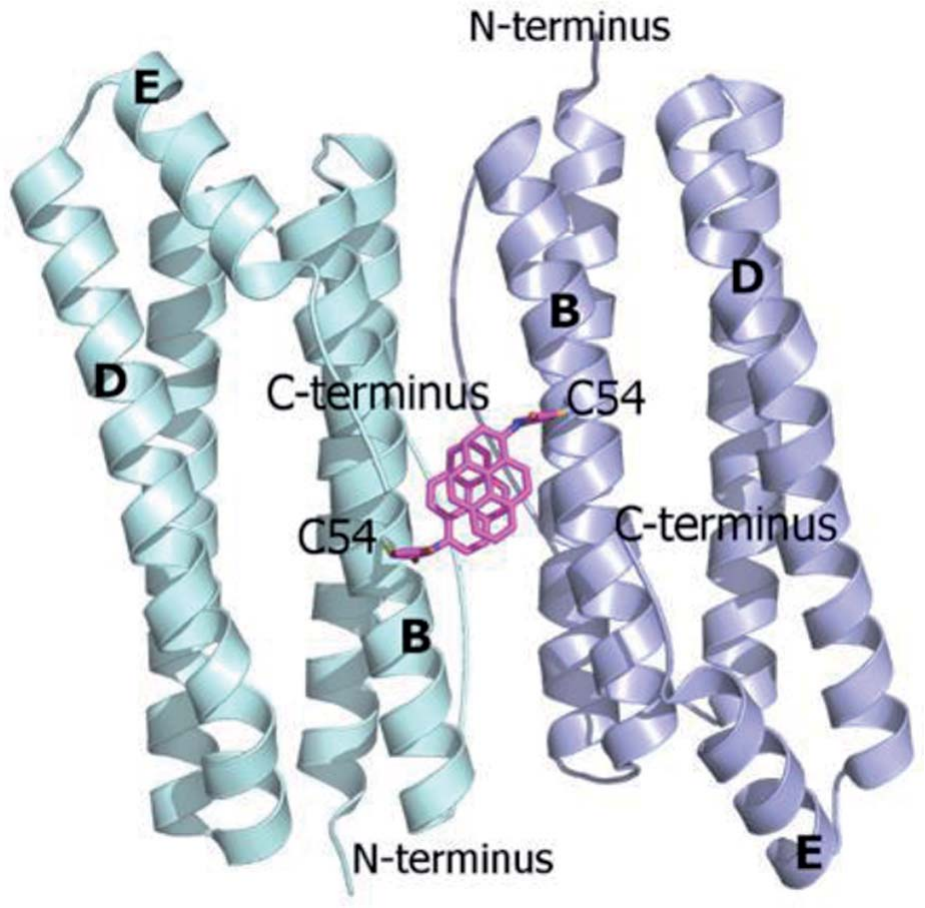
Ribbon diagram of the antiparallel homodimer structure of HumAfFt (pdb 5LS9). Two NPM molecules are depicted in magenta sticks bound to C54, establishing a π–π stacking interaction, at the dimer interface.

Ferritins are cage-like proteins with a unique tetraeicosameric (24-meric) assembly that usually displays a highly conserved basic unit, a monomer, composed of a four-helix bundle namely A, B, C and D, and a short E helix at the C-terminus.^29a,b^ In high NaCl and MgCl_2_ concentrations AfFt and chimeric ferritin HumAfFt assemble in their 24-meric cage-like shape while, in the absence of ions, the four-helix bundle of both ferritins is found as a stable antiparallel dimer.^11,12,30,31^ At the two-fold axis of the dimer, in the middle of the B helix, a single point conservative mutation (M54C) was introduced for site-selective *N*-(1-pyrenyl)maleimide (NPM) labeling. The mutated residues were positioned far from any inter-subunit contacts or loop regions in order to avoid any interference with the cage assembly, at a 14 Å distance between two β-carbons of two C54, enough for a π-stacking interaction between two pyrene molecules (Fig. 1). As previously demonstrated, the overall fold and function of HumAfFt, in comparison with AfFt, were not altered by the amino acid substitution.^12^ Hereafter, we will refer to HumAfFt and AfFt as both mutated at position 54.

In addition, a mutant of *Pyrococcus fulgidus* ferritin (PfFtP77C) was designed with a cysteine residue on the external surface for NPM labeling and used as pyrene monomer fluorescence standard, whereas a distance of 24 Å between two cysteines did not allow for excimer formation.^15^ Moreover, as PfFtP77C was uniquely found as a 24-meric assembled cage in solution, the protein was used as a standard independent of cation concentration.^32^

### 2.2 Pyrene-labeled ferritin preparation

Optimal experimental conditions for the conjugation reaction between ferritins and NPM were extensively described in the ESI† and the successful conjugations were confirmed by MALDI-TOF Mass Spectrometry analysis. Indeed, the obtained molecular weights of 20 458 Da for pyrene-labeled AfFt and 20 545 Da for pyrene labeled-HumAfFt (Fig. S1†) were in agreement with the predicted ones. The labeling percentage was determined from the free-sulfhydryl content obtained by averaging the Ellman's reagent absorbance at 412 nm from three samples, since a UV-Vis determination based on the pyrene absorption peaks was not reliable due to red-shift and hypochromic effect caused by micro-environment changes.^25^

### 2.3 Ferritin's assembly assessment by DLS

Dynamic light scattering (DLS) experiments were carried out to confirm that ferritin shape and oligomerization properties were not altered by pyrene-labelling.

The hydrodynamic diameters of both pyrene-labeled HumAfFt and AfFt proteins showed similar size to the respective native ferritins and were found to be monodispersed in solution. The hydrodynamic diameters measured by DLS experiments were 5.8 nm for the dissociated state in the absence of MgCl_2_, in agreement with the predicted theoretical value for a dimer,^33^ and approximately 14 nm for the associated state in 20 mM MgCl_2_ (Fig. S2†).

The overall oligomerization process was unchanged by pyrene-labeling as the Mg^2+^-dependent titrations confirmed the comparable self-assembly properties of AfFt and HumAfFt, with and without pyrene (Fig. S3†).

### 2.4 Ferritin's assembly-disassembly assessment by fluorescence spectroscopy

**Fig. 2:**
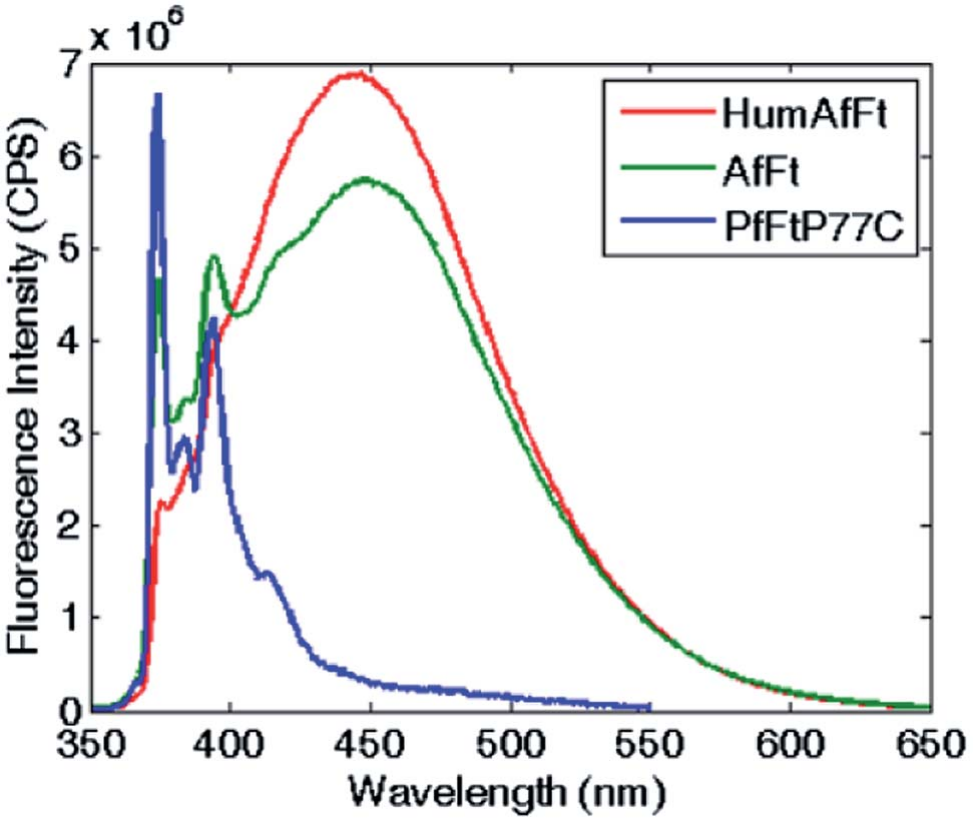
Fluorescence emission spectra of pyrene-labeled ferritins. The characteristic pyrene–monomer peaks in PfFtP77C are shown in blue. In AfFt and in HumAfFt, the excimer emission profiles shown in green and red, respectively.

The self-assembly process of AfFt and HumAfFt ferritins was finally extensively studied by exploiting pyrene characteristic fluorescence emission, while PfFtP77C was employed as assembled 24 meric cage and pyrene monomer standard. As shown in Fig. 2, only the pyrene monomer fluorescence emission was present in PfFtP77C, while in AfFt and HumAfFt an additional broad band appeared, thus confirming the excimer formation. Surprisingly a higher amount of excimer was found in HumAfFt although this protein differed from AfFt by only 9 residues on the loop between helixes B and C and the superposition of the two structures did not highlight any significant repositioning in the residue 54. For an efficient excimer formation, two pyrene molecules must be stacked in a parallel orientation allowing for a π–π interaction to take place, and these mutations on the loop could possibly slightly affect the relative orientation and distance between the two pyrene moieties.^15^

**Fig. 3:**
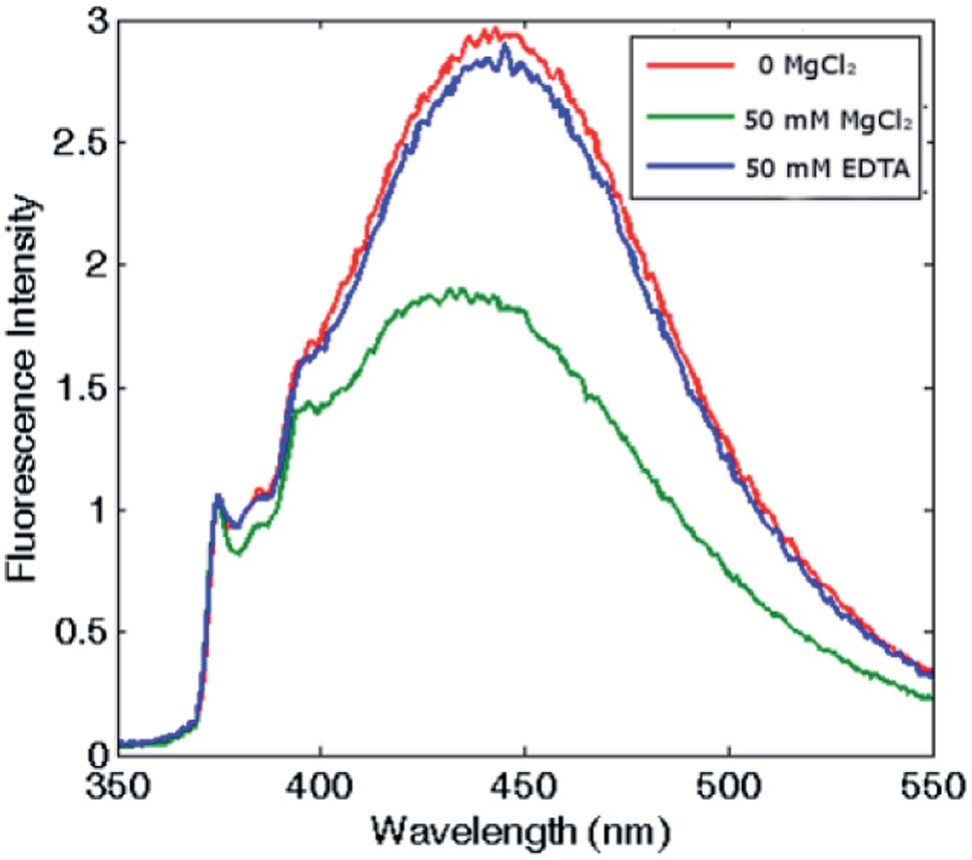
Reversibility assessment of the assembly-disassembly mechanism. Fluorescence spectra of pyrene–HumAfFt in the absence of salts (red), in the presence of 50 mM MgCl_2_ (green) and after 50 mM EDTA addition (blue). The excimer content difference in presence or absence of MgCl_2_ reflects ferritin's association state.

Remarkably, in the tetraeicosameric state, the overall amount of excimer decreased, as shown in Fig. 3 and S4,† probably due to movements at the inter–dimer interface that cause a destabilization of π-stacking interactions between the two pyrene moieties. Moreover, the excimer amount was successfully restored after EDTA addition, confirming that the chelating ability of this agent promoted protein disassembly by removal of magnesium cations (Fig. 3, Fig. S4†).

**Fig. 4:**
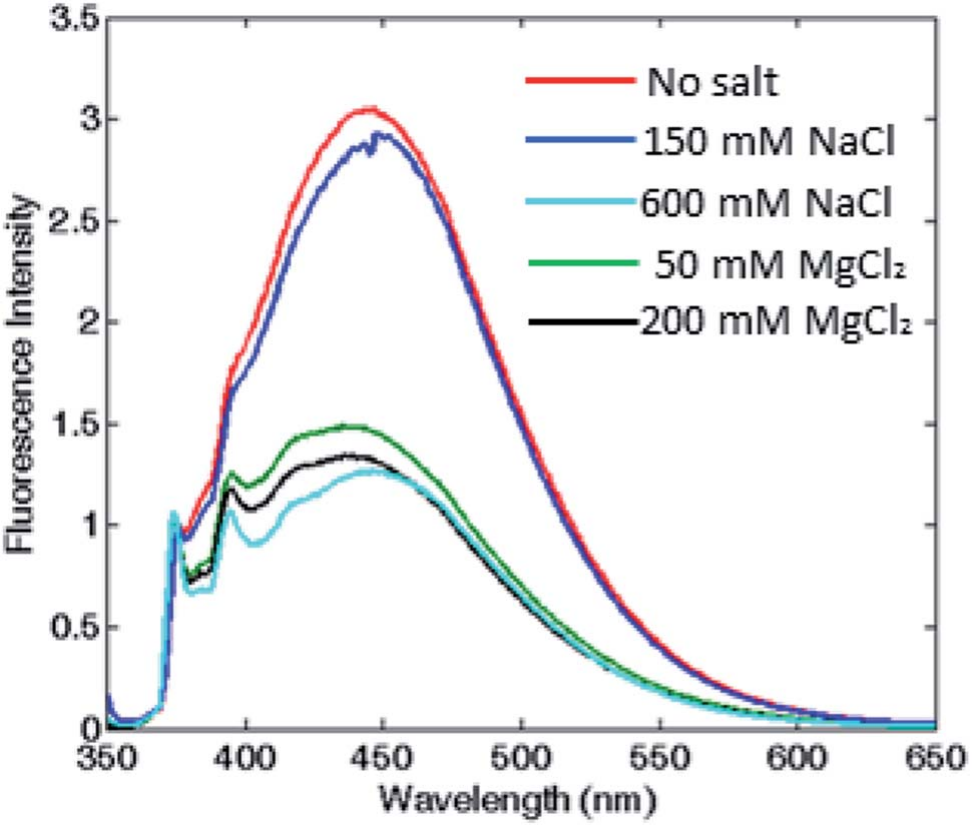
Assessment of the correlation between oligomerization state and excimer fluorescence. Fluorescence emission spectra of HumAfFt, without any salt and at two different fixed ionic strengths for either MgCl_2_ or NaCl. Spectra in the absence of any salt (red) and 150 mM NaCl (blue) correspond to a dimeric state, while the profiles at 600 mM NaCl (cyan), 50 mM (green) and 200 mM (black) MgCl_2_, correspond to the associated state.

To corroborate that the fluorescence excimer broad band variation was actually related to the oligomerization process and was independent from the ionic strength, we measured fluorescence emission at fixed ionic strength using different concentrations of either MgCl_2_ or NaCl. According to the Debye–Hückel model, the experiment was carried out at two different ionic strength conditions: at low salt concentrations (*i.e.* 150 mM NaCl or 50 mM MgCl_2_), where the protein was associated in the 24-meric state only in MgCl_2_, and at high salt concentrations (*i.e.* 600 mM NaCl or 200 mM MgCl_2_), where the protein was known to be associated in both salts.^11,12,30,31^ As shown in Fig. 4 and S5,† in the first condition, the excimer content without salt and at 150 mM NaCl were similar, in agreement with a dissociated state, whereas at 50 mM MgCl_2_, therefore at equal ionic strength, the excimer content revealed the presence of an associated state for both proteins. Moreover, the pyrene monomer emission profile of PfFtP77C did not change at various salt concentration, excluding any ionic strength influence on the pyrene fluorescence emission (data not shown).

**Fig. 5:**
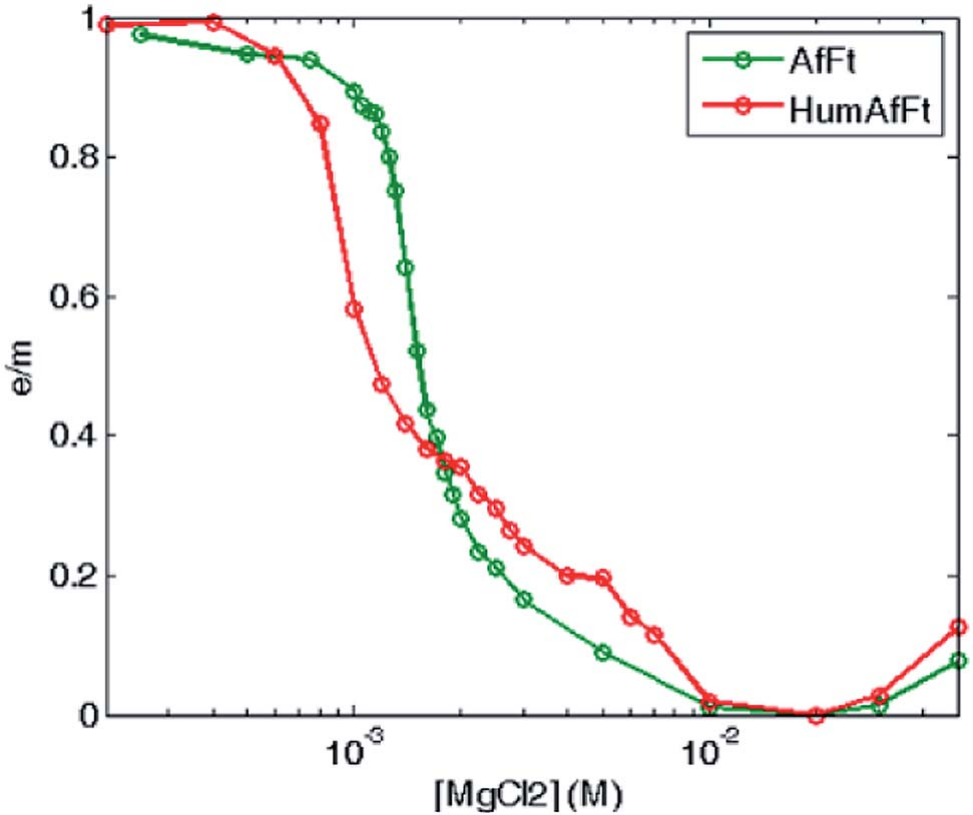
Thermodynamic study of Mg^2+^-triggered oligomerization process. Fluorescence e/m ratio is shown as a function of magnesium concentration, at equilibrium at 25 °C, for pyrene-labeled HumAfFt (red) and AfFt (green) highlighting a highly cooperative transition.

Fluorescence emission dependence on the protein oligomerization state was thus investigated by monitoring magnesium induced association at equilibrium. A sigmoidal curve was obtained by plotting excimer/monomer (e/m) ratio as a function of MgCl_2_ concentration, suggesting a strong cooperativity within the association process, with a complete assembly at about 5 mM MgCl_2_ in both ferritins (Fig. 5).

The strong effect of Mg^2+^ ions on subunit assembly was then analyzed in terms of possible contributions to specific binding within the three-dimensional crystallographic structure (pdb 5LS9) by using PISA software (CCP4 suite). No significant evidence of Mg^2+^ presence at the interface between dimers was observed. In fact, as it was previously reported,^11,34^ it was evident that the hydrophobic network connecting the dimers' interface played a key role in stabilizing the cage structure in high ionic strength buffers to minimize the solvent exposure. In turn, in the X-ray structure, Mg^2+^ cations were found to bind the ferroxidase center through explicit coordination provided by Asp52, Glu19 and His55, according to HumAfFt numbering, thus strengthening the Sana's assumption that divalent cations occupy and competitively inhibit iron binding under conditions of iron deficiency.^34,35^ In the light of these observations, it might be hypothesized that coordination of Mg^2+^ in the ferroxidase center can therefore assist the assembly process by exerting some subtle conformational rearrangement within the dimeric species, thus explaining the different behavior respect to a monovalent cation which is prevented from binding.

### 2.5 Ferritin's assembly kinetics

In order to determine the kinetics of the salt-induced oligomerization process, stopped-flow experiments were carried out by monitoring fluorescence changes over time. As the total fluorescence emission was higher for the disassembled ferritin, a fluorescence decrease over time was expected during protein oligomerization.

**Fig. 6:**
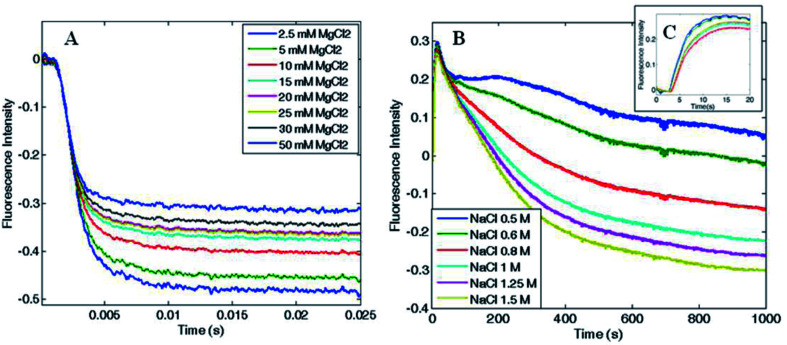
Association kinetics as a function of (A) MgCl_2_ (B and C) and NaCl concentration. HumAfFt kinetic rates were recorded at (A) 4 °C increasing MgCl_2_ concentrations showing a fast biexponential behavior complete in 25 ms, and at (B) 25 °C increasing NaCl concentrations showing a slow multi-phasic behavior barely complete after 1000 s. In the close-up view, (C), the initial lag phase is shown (≤5 s).

Magnesium-triggered assembly was too fast (time scale < 10 ms) to be followed by stopped-flow measurements at 25 °C. However, it was possible to single out a clear decay curve at lower temperature (4 °C). Despite the assembly occurred close to the instrumental time limit, some semi-quantitative estimates on Mg^2+^-induced association could still be obtained. Fluorescence emission decreased with a double exponential trend and the reaction was complete within 25 ms, as shown in Fig. 6A. Reaction rates increased proportionally with both Mg^2+^ and protein concentration with a major contribution from the protein concentration, which showed an apparent second order rate constant of about 10^6^ M^−1^ s^−1^. In contrast, the EDTA-triggered dissociation process was slower than the assembly and was measured both at 25 and 4 °C. Faster dissociation rates were observed at higher temperature, with completeness reached in 0.1 s at 25 °C and in 0.25 s at 4 °C. Dissociation showed a biphasic profile, dependent on EDTA concentration but independent of protein concentration (Fig. S6†), as expected.

Since the kinetic reaction in the presence of magnesium cations appeared to be too fast, we investigated the slower NaCl induced association process. In comparison with magnesium, higher salt concentrations were required to monitor Na^+^-triggered association as the oligomerization was complete only at 500 mM NaCl.^30^ The assembly showed much slower rates and a multiphasic kinetic profile (Fig. 6B) with an initial lag phase (Fig. 6C), typical of a nucleation process commonly found in protein oligomerization,^36^ that evolved in four exponential phases toward the end of the reaction, which was reached only after 20–30 minutes at 25 °C. These changes in the total fluorescence emission over time, could be due to multiple pyrene rearrangement during the assembly. Additionally, while the association rates were proportionally increasing with NaCl concentration, at fixed NaCl concentration and varying protein concentration the reaction rates were approximately constant. These evidences documented the complexity of the aggregation mechanism and suggest that a different pattern might be involved in the magnesium-triggered association compared to the sodium-triggered one. Previous work on the mechanism of self-assembly of ferritins is limited to only a few set of experimental investigations. The pH-triggered reassembly of apoferritin from horse spleen, has been followed by means of sedimentation velocity and circular dichroism experiments.^37^ This work suggested that dimers, tetramers (or trimers) and octamers (or hexamers) are in rapid equilibrium on the ultracentrifuge time scale and that the assembly process is complex and dependent on buffer type and the presence of chloride and phosphate. In other work,^38^ the kinetics of reassociation of horse spleen ferritin were followed by intrinsic protein fluorescence, circular dichroism and chemical crosslinking which essentially confirmed the sequential nature of the assembly process in agreement with Stefanini *et al.* (1987).^37^ Finally a more recent investigation, that made use of time-resolved small angle X-ray scattering successfully monitored the pH-induced oligomerization of *Escherichia coli* ferritin on a time scale from milliseconds to minutes.^14^ These experiments strongly suggest of significant formation of intermediate oligomers during the assembly reaction, although in the proposed mechanism third-order reactions were included to fit the data.

The present experiments are a first step in unraveling the details of the aggregation process of ferritins by using a simple spectroscopic technique. Protein–protein association rate constants span a quite large range of values from 10^3^ to over 10^9^ M^−1^ s^−1^.^39^ From Fig. 6A the halftime of the reaction is ∼1–2 ms it may be roughly estimated that the second-order rate constant is in excess of 10^8^ M^−1^ s^−1^ at 4 °C, and larger at higher temperatures, when using Mg^2+^ as the triggering ligand. Thus, the structural determinants that confer the large stability of the ferritin tetraeicosamer is likely due to the fast association constants of the ferritin polypeptides.

### 2.6 Visualization of pyrene-labeled ferritins by two photon fluorescence microscopy

Preliminary analysis to study the behaviour of the pyrene fluorophore in its excimeric and monomeric states by two photon fluorescence microscopy were performed by comparing pyrene-labelled HumAfFt and pyrene-labelled PfFt P77C absorption and emission curves through single photon (Fig. S7A and C†) and two photon spectroscopy (Fig. S7B and D†). As reported in the Fig. S7,† the overall spectral behavior was indeed preserved in the single and two photon approach, even in the presence of slight differences due to the different absorption/emission cross sections of the electronic processes involved, as observed in several experimental studies.^40a,b^

A careful analysis of the monomer and excimer spectra showed that, independently of the excitation wavelength, their spectral behaviour was remarkably different, as a further confirmation of the excimer formation in the ferritin cage-like structure.

### 2.7 Visualization of pyrene–HumAfFt-TRITC inside HeLa living cells by two photon fluorescence microscopy

**Fig. 7:**
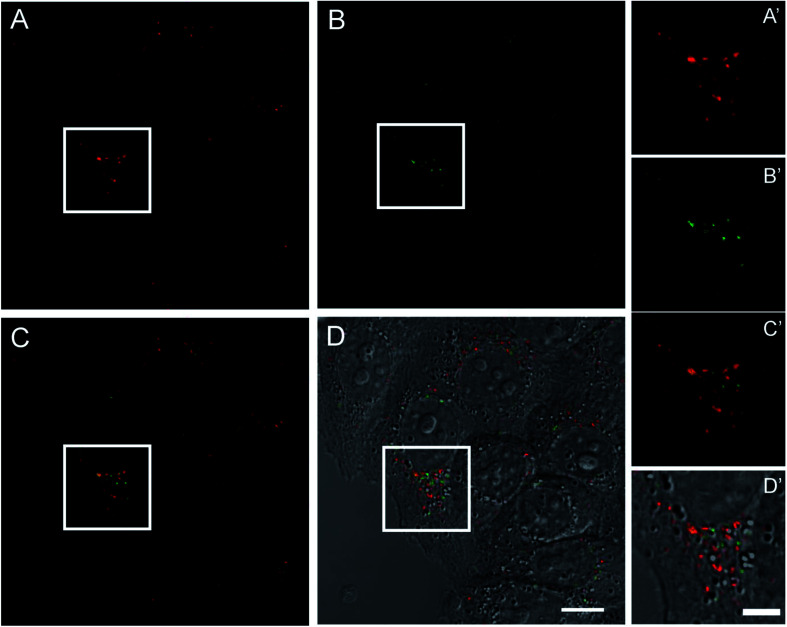
Confocal imaging of living HeLa cells incubated with pyrene–HumAfFt-TRITC. Images were acquired at one and two-photon excitations to detect respectively the TRITC (A) and the pyrene excimer emission (B). Merged signals and the overlay with the DIC image are shown in C and D, respectively (scale bar is 10 μm). Magnified images of the highlighted regions are showed in corresponding panels A′, B′, C′ and D′(scale bar is 5 μm).

To evaluate the performance of this novel nano-device as active fluorescent probe, living HeLa cells expressing high levels of TfR1 receptor, similar to many tumor cells, were incubated with pyrene-labeled HumAfFt-TRITC and imaging studies were carried out by TPFM. The HumAfFt internalization process was not altered by pyrene-labeling as confirmed by TRITC signal, detected with one photon excitation (Fig. 7A and A′), distributed in the cytoplasm and in the perinuclear space as expected.^10^ In the same cells, switching to TPFM imaging, we were able to detect pyrene excimer emission in the same locations (Fig. 7B and B′) and a partial co-localization was highlighted (Fig. 7C–D′). The partial overlap between pyrene and TRITC signals could be explained by two main aspects. First, the two imaging techniques had intrinsic different integrated z-section, smaller in the TPFM compared to confocal imaging, meaning less particles visible in a single plane. Second, in the delay time during the switching mode between one and two photon excitation, the rearrangement in the intracellular compartments of living cells occurred.

## 3. Experimental section

### 3.1 Protein production

The chimeric HumAfFt ferritin was designed and produced as previously reported and its assembly properties were verified by size exclusion chromatography (SEC).^12^ The mutated ferritin from *Pyrococcus furiosus* PfFtP77C and *Archaeoglobus fulgidus* AfFtM54C, already available in our lab, were purified as previously reported.^31,41^ Protein concentrations were measured by UV spectra using a molar extinction coefficient of 31 400 M^−1^ cm^−1^ for PfFtP77C, 33 900 M^−1^ cm^−1^ for AfFtM54C and 32 400 M^−1^ cm^−1^ for HumAfFt.

### 3.2 Pyrene-labeled ferritin preparation

Ferritins (4 mg ml^−1^, in 20 mM HEPES pH 7.4, 50 mM MgCl_2_) were reduced with TCEP (10 eq.) for 1 h at room temperature under mild agitation. The conjugation reaction was carried out in 15% v/v acetonitrile to favor NPM solubility. The commercially available *N*-(1-pyrenyl)maleimide linker (Sigma Aldrich, 5 eq.) was slowly added to the protein solution and the reaction mixture was left under stirring for 4 hours at 37 °C. NPM excess was removed by gel filtration chromatography (G25 Desalting, eluent 20 mM HEPES pH 7.4, 15% v/v acetonitrile), co-solvent was removed by dialysis against 20 mM HEPES pH 7.4 and, in order to remove excess MgCl_2_ and maintain the protein in its dimeric state, 5 mM EDTA was added to the dialysis buffer. Extensive dialysis against 20 mM HEPES pH 7.4 was then performed.

Determination of free sulfhydryl was performed on a UV5600 Spectrophotometer using Ellman's assay according to standard procedure. Briefly, a stock solution of 5,5-dithio-bis-(2-nitrobenzoic acid) (Ellman's reagent) was mixed with the protein sample in a 20-fold molar excess. The solution was incubated for 5 minutes at room temperature and UV absorption was measured at 412 nm, using as blank, a sample containing the same amount of Ellman's reagent in buffer. The sulfhydryl per protein ratio (SPR) was calculated from the ratio between the Ellman's reagent concentration (*ε* = 14 150 M^−1^ cm^−1^) and the protein concentration. Every experiment was repeated 3 times and the results were calculated based on the average of the 3 experiments.

### 3.3 Dynamic light scattering measurements

DLS experiments were carried out with a Zetasizer Nano S (Malvern Instruments, Malvern, UK.) equipped with a 4 mW He–Ne laser (633 nm). Measurements were performed at 25 °C, at an angle of 173° with respect to the incident beam. All samples were prepared at 2 mg ml^−1^ in HEPES 20 mM pH 7.4 and various MgCl_2_ concentrations, and were filtered with a 0.2 μm filter before analysis. Peak intensity analysis was used to determine the average hydrodynamic diameters (*Z*-average diameter) of the scattering particles.

### 3.4 Fluorescence spectroscopy measurements

Fluorescence measurements were carried out with a FluoroMax 4 (Horiba) spectrofluorimeter with a Haake D8 refrigerated bath at 25 °C and at equilibrium. Fluorescence emission spectra of pyrene-labeled samples (30 μM in 20 mM HEPES pH 7.4) were excited at 342 nm and fluorescence data were collected from 350 to 650 nm. The association process dependence on ionic strength was investigated varying concentrations of MgCl_2_ and NaCl, while the dissociation mechanism was studied by EDTA treatment. Emission spectra were registered 5 minutes after each MgCl_2_ and EDTA addition and after 30 minutes for any NaCl addition, until no further changes in the fluorescence spectra were detected, and were normalized with respect to the maximum absorption peak of a single pyrene molecule and the excimer/monomer ratio, calculated as the fluorescence intensity of the excimer peak (445 nm)/monomer peak (375 nm) ratio, was used as a relative indicator of the extent of excimer formation.

### 3.5 Stopped-flow measurements

Kinetic measurements were carried out on a single mixing stopped-flow apparatus (Applied Photophysics, Leatherhead, UK) at 4 °C and 25 °C. Samples were excited at 342 nm (6 nm slits) and total emission fluorescence was collected with a 360 nm long-pass filter.

Association kinetic experiments were performed at 5 μM protein before mixing (b.m.) in 20 mM HEPES pH 7.4, increasing concentrations of MgCl_2_ or NaCl and, at constant salt concentration (either 20 mM MgCl_2_ or 2 M NaCl b.m.) increasing protein concentrations (5 to 100 μM ferritin b.m.). Dissociation kinetic experiments were performed at 5 μM protein b.m. in 20 mM HEPES pH 7.4 and MgCl_2_ increasing EDTA/Mg^2+^ molar ratios or, at a fixed EDTA concentration (20 mM) increasing protein concentrations (5 to 100 μM ferritin in 20 mM MgCl_2_ b.m.).

### 3.6 Pyrene-ferritin-TRITC preparation, cell culture, ferritin internalization and visualization into HeLa cells

Pyrene-ferritins were further labeled with TRITC (tetramethylrhodamine isothiocyanate, Thermofisher) following manufacturer's standard protocol. HeLa cells were grown at 37 °C in DMEM without phenol red and supplemented with 10% (v/v) FBS, Glutamax (Invitrogen) and penicillin–streptomycin solution (Sigma). The internalization assay was performed as follows: cells were seeded on the 8-well μ-slide ibiTreat (ibidi) and after 24 h, TRITC-ferritin nanoparticles (pyrene–HumAfFt-TRITC and HumAfFt-TRITC as control) were added at the final concentration of 300 μg ml^−1^ and incubated for 48 h. Just before imaging, cells were washed to eliminate the unbound ferritin and then acquired by confocal laser-scanning microscopy.

## 4. Conclusions

In this paper, the pyrene excimer emission was exploited to provide new kinetic and thermodynamic insights on the archaeal ferritins oligomerization mechanism, and it was employed for a two photon excimer-based intracellular visualization of pyrene-labeled proteins.

Our results clearly demonstrate the strong correlation between pyrene excimer fluorescence and ferritin oligomerization state, thus highlighting the importance of this pyrene-based method as a powerful tool through which easily gain insights into the thermodynamic and kinetics details of protein oligomerization mechanism. Indeed, while both mono and divalent cations were capable of triggering ferritin association, divalent cations showed very fast kinetics, within a millisecond time scale, and were able to completely form the 24-meric cage at low MgCl_2_ concentrations, thus potentially representing a convenient alternative to NaCl for the synthesis of drug-delivery nano devices. The oligomerization process was also confirmed to be remarkably cooperative and fully reversible by EDTA treatment. Furthermore, pyrene–HumAfFt was highly internalized by HeLa cells and pyrene excimer displayed an intense fluorescence emission by two photon fluorescence confocal microscopy, which was comparable to common fluorophores emission such as TRITC. The pyrene-based bioconjugate system confirmed to be an excellent building block for two photon fluorescent nano-particle design, which could be further optimized for diagnostic and biomedical application based on the ability of HumAfFt to target the overexpressed TfR1 receptor in human cancer cells.

## Conflicts of interest

There are no conflicts to declare.

## Abbreviations

NPM: *N*-(1-Pyrenyl)maleimide
TPFM: Two photon fluorescence microscopy
TCEP: Tris(2-carboxyethyl)phosphine
EDTA: Ethylenediaminetetraacetic acid

## Supplementary Material

RA-008-C8RA00210J-s001
