# Supplementary material for: Excimer based fluorescent pyrene–ferritin conjugate for protein oligomerization studies and imaging in living cells
Source: RSC Adv. 2018 Apr 3;8(23):12815–22. doi: 10.1039/c8ra00210j (PMC9079363; doi:10.1039/c8ra00210j)
Supplement: RA-008-C8RA00210J-s001 [file RA-008-C8RA00210J-s001.pdf]

Electronic Supplementary Material (ESI) for RSC Advances.
This journal is © The Royal Society of Chemistry 2018

SUPPORTING INFORMATION

Excimer based fluorescent pyrene-ferritin conjugate for protein oligomerization studies and imaging in living cells

Authors: Irene Benni[a], Matilde Cardoso Trabuco[a,b], Enrico Di Stasio[c], Alessandro Arcovito[c], Alberto Boffi[d,e], Francesco Malatesta[a], Alessandra Bonamore[a], Simone De Panfilis[e], Valeria de Turris[e], Paola Baiocco[e]*

Affiliations:

a) Department of Biochemical Sciences "Alessandro Rossi Fanelli", Sapienza University of Rome, P.le A. Moro 5, 00185 Rome, Italy

b) Molirom srl, via Ravenna 8, 00161 Rome, Italy

c) Institute of Biochemistry and Clinical Biochemistry, Catholic University, Largo Francesco Vito, 1, 00168 Rome, Italy

d) Institute of Molecular Biology and Pathology, National Research Council, P.le A. Moro 7, 00185 Rome, Italy

e) Center for Life Nano Science@Sapienza, Istituto Italiano di Tecnologia, V.le Regina Elena 291, 00161Rome, Italy

SUPPLEMENTARY EXPERIMENTAL SECTION

1.1 Pyrene-labeled ferritin preparation

Optimization of ferritin labeling reaction was extensively carried out screening reaction conditions such as temperature, reagent equivalents, protein concentration and co-solvent presence. Due to the hydrophobicity and the aromatic nature of pyrene, the presence of a co-solvent in the reaction mixture, DMSO or acetonitrile, proved to be crucial. However, a compromise between NPM solubility and protein stability in co-solvent had to be found. The best co-solvent resulted to be acetonitrile but, even in the presence of co-solvent, the NPM reagent couldn't be added in higher amounts than 5 excesses without protein loss due to precipitate formation. The chosen conditions afforded a labeling percentage of 75 % and a protein recovery of 85 %.

Control to assess possible unspecific interactions of NPM with ferritin hydrophobic regions was carried out adding NPM, under identical reaction conditions to those previously described, to the AfFt wild type protein not bearing any cysteine residue. To this end, purification with a co-solvent resulted to be crucial and, adjusting purification conditions, the unspecific hydrophobic attachment was reduced to approximately 5 %.

1.2 Mass spectrometry measurements

Each sample was desalted on C8 Empore Disk (3M, Minneapolis, MN) home-made stage tip and resuspended in 3 μl formic acid 1%. 1μl was spotted on a MALDI sample plate and allowed to air

dry. Recrystallized sinapinic acid (SA matrix from Thermo Fisher Scientific) was prepared at a concentration of 5 mg/ml in 50:50 acetonitrile/water (0.1% FA) and spotted directly prior to insertion into the mass spectrometer. Matrix-assisted laser desorption ionization (MALDI) mass spectra were acquired on 4800 MALDI-TOF/ TOF mass spectrometer (Applied Biosystems, Foster City, CA) equipped with a nitrogen laser operated at 336 nm laser. Acquisitions were performed in linear mode averaging 2500 laser shots in a random, uniform pattern. Ions were accelerated with a 20 kV pulse, with a delayed extraction period of 860 ns. Spectra were generated by averaging between 500 and 2000 laser pulses in a mass range from 4KDa to 50 KDa. Laser intensity was set to optimize the signal-to-noise ratio and the resolution of mass peaks of the analyte. All spectra were externally calibrated and processed via Data Explorer (version 4.7) software.

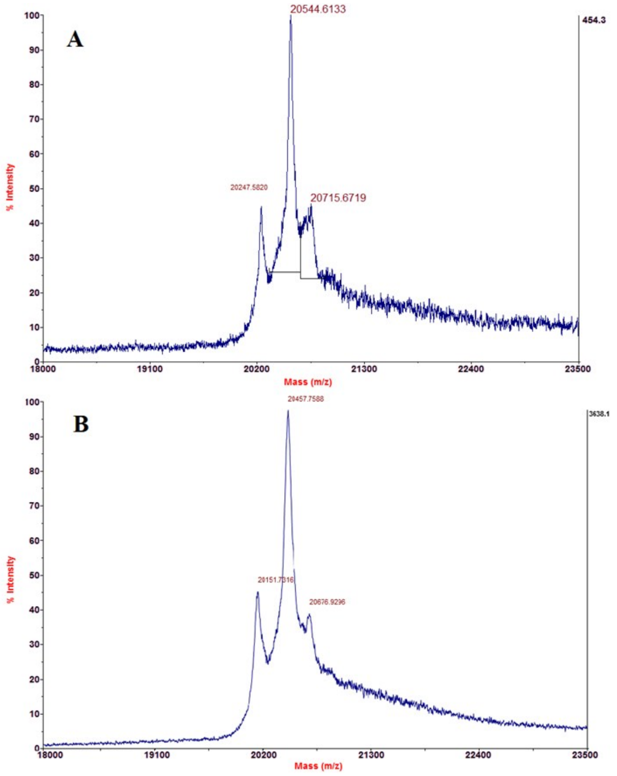

Figure S1: MALDI-TOF Mass spectrometry analysis.
Pyrene conjugate to: A) HumAfFt, where the peaks correspond to the protein without the first methionine residue (mass observed 20248 Da, expected 20243 Da) and to the NMP-HumAfFt conjugate (mass observed 20545 Da, expected 20540 Da) and B) AfFt, where the peaks correspond to the protein without the first methionine residue (mass observed 20152 Da, expected 20157 Da) and to the NMP-AfFt conjugate (mass observed 20458 Da, expected 20454 Da).

1.3 Dynamic light scattering measurements

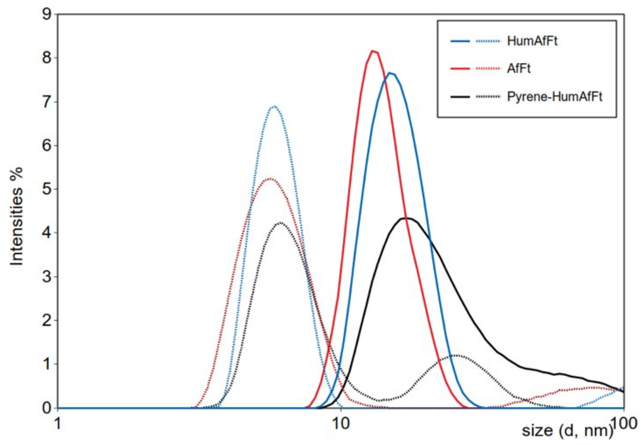

Figure S2: DLS experiments

DLS measurements for pyrene-labeled HumAfFt (black) are shown in 20 mM Hepes, 50 mM $MgCl_2$ as dashed lines, and in 20 mM Hepes, pH 7.4 as continued lines. DLS experiments confirmed the similar size and the oligomerization properties of the pyrene-labeled HumAfFt in comparison with HumAfFt (blue), AfFt (red).

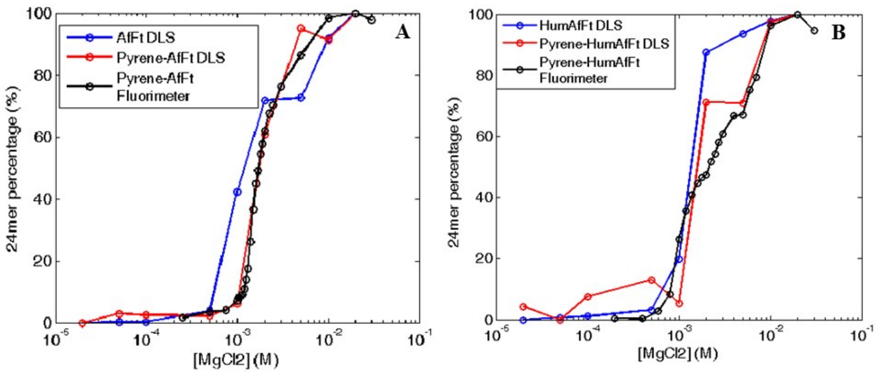

Figure S3: DLS titration compared with fluorescence titration as a function of Mg concentration.

DLS measurements for (A) AfFt and (B) HumAfFt, native (blue) and pyrene-labeled (red), are compared with fluorescence measurements on pyrene-labeled ferritins (black). All the measurements show a cooperative association not altered by pyrene labeling.

1.4 Fluorescence spectroscopy measurements

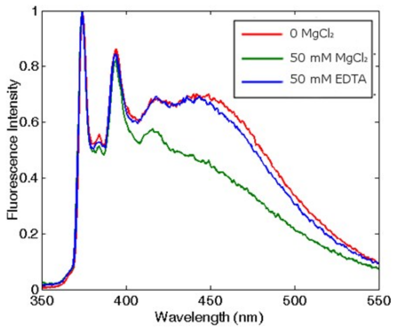

Figure S4: Reversibility assessment of the assembly-disassembly mechanism.
Fluorescence spectra of pyrene-AfFt in the absence of salts (red), in the presence of 50 mM $MgCl_2$ (green) and after 50 mM EDTA addition (blue). The excimer content difference in presence or absence of $MgCl_2$ reflects ferritin's association state.

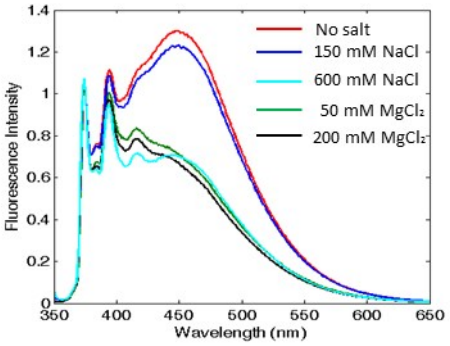

Figure S5: Assessment of the correlation between oligomerization state and excimer fluorescence.
Fluorescence emission spectra of AfFt, without any salt and at two different fixed ionic strengths for either $MgCl_2$ or NaCl. Spectra in the absence of any salt (red) and 150 mM NaCl (blue) correspond to a dimeric state, while the profiles at 600 mM NaCl (cyan), 50 mM (green) and 200 mM (black) $MgCl_2$, correspond to the associated state.

In order to exclude the hypothesis that the excimer band arised from intermolecular interactions due to small unspecific NPM binding, additional fluorescence measurements were carried out. Fluorescence spectra were recorded diluting the sample several times and the e/m ratio was analysed. As expected, the total florescence decreased but the e/m ratio never varied demonstrating that the excimer emission is only due to intramolecular interactions of two closed pyrene molecules (data not shown).

1.5 Stopped flow measurements

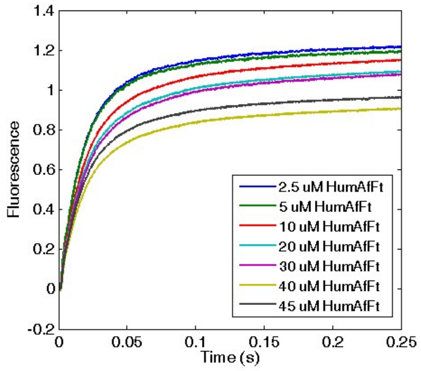

Figure S6: Dissociation kinetics of Pyrene-HumAfFt as a function of protein concentration. HumAfFt kinetic rates were recorded at 4 °C increasing protein concentrations at a fixed EDTA/$MgCl_2$ ratio.*1.6 Two photon and confocal fluorescence microscopy measurements on HeLa cells*

Microscope images were acquired through an inverted Olympus IX83 microscope (Olympus Europe, Hamburg, Germany), equipped with either an UPLSAPO 10X2 objective (N.A. 0.4) for spectral measurements of pyrene-labeled ferritins or an UPLSAPO 60X water immersion objective (N.A. 1.2) for HeLa cell uptake imaging. Confocal aperture was 80 micron and 120 micron respectively, for a corresponding theoretical resolution of 680x680 nm and 220x220 nm (HxV). The collinear light beams from a 559 nm laser diode light source and a 690-1040 nm tunable Spectra-Physics Mai Tai DeepSee Ti:Sapphire pulsed laser source were injected into the microscope via a FV1200 MPE laser scanning confocal device. A set of adjusted dichroic mirrors plus a band-pass grating filter (for the TRITC channel) and a 405-540 nm emission filter (for the non-descanned photon detection) deflected the fluorescence emitted radiation into photomultiplier (PMT) detectors for integrated light intensity measurements. An optical condenser (0.55 NA) collected the transmitted radiation to the bright-field image detector. The PMT high-voltages were adjusted such that no pixels were saturated in the images. The 1024x1024 pixel fluorescence images (1270.6x1270.6 nm, 10X2 objective, and 70.7x70.7 nm, 60X objective, field of view, respectively) were collected in line sequential mode. Z-stacks were collected at 200 nm slice interval, for a total counting time of about 300 s per series.

1.7 Two-photon fluorescence spectroscopy measurements

Preliminary two-photon fluorescence microscopy measurements were carried out on pyrene-labeled PyFtP77C, pyrene-labeled HumAfFt and the unlabeled proteins as control, at a 416 µM concentration. Each sample was injected into the channels (90.9 µm thick and 90.9 µm wide) of a polymethylsiloxane microfluidic device and illuminated with the tuneable IR light from the pulsed source at a constant integrated power of 500 mW over the entire range of frequencies. The emitted

fluorescence from the separated channel device was integrated in the 405-540 nm interval by a PMT detector and elaborated with Fiji software. Fluorescence emission of pyrene excimer and monomer, corresponding to pyrene-labeled HumAfFt and PyFtP77C respectively, were recorded using the unlabeled ferritin signal as blank (Figure S6B).

The spectral distribution of the fluorescence signal in the UV-Visible range at a 755 nm multiphoton excitation wavelength, was measured by a PI Acton SpectraPro SP-2300 spectrometer equipped with a 150 gr/mm horizontal diffraction grating and a PI Pixis 256 CCD camera. The emitted radiation was collected by a 665 nm cut dichroic mirror placed in a NDD adapter from PicoQuant and coupled to the spectrometer by a liquid light guide and collection optics. A further short pass filter (Omega Optical 630SP RapidEdge) next to the spectrometer entrance slit reduced the IR excitation line by about 7 orders of magnitude. The spectral images onto the CCD camera were vertically rebinned and integrated for 100 s. Eventually, the background from unlabeled ferritin emission was subtracted to provide the spectra shown in Figure S6D.

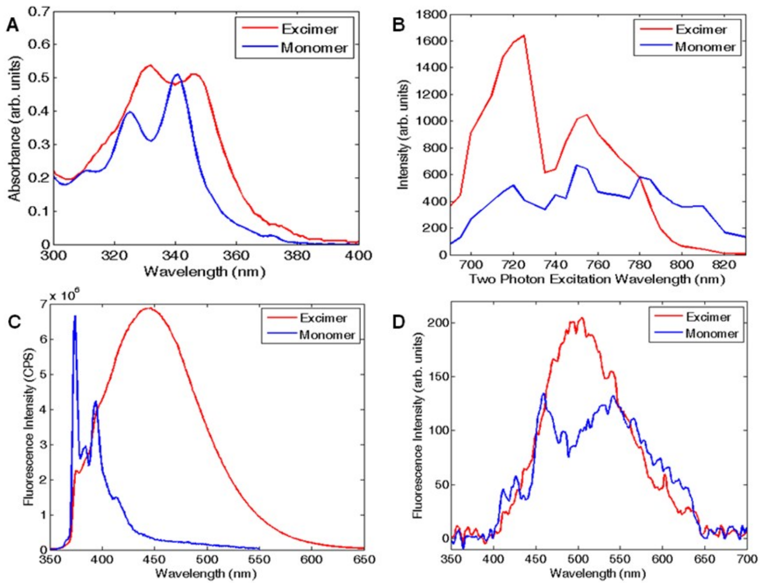

Figure S7: Pyrene excimer and monomer absorption and emission curves.
In all panels, pyrene-labeled PyFtP77C and pyrene-labeled HumAfFt corresponding to monomer and excimer pyrene are shown in blue and red respectively: A) UV-Vis absorption spectra, B) Spectral integrated fluorescence as a function of the two-photon exciting wavelength, C) Fluorescence emission spectra recorded exciting at 342 nm, D) Two-photon fluorescence emission spectra recorded exciting at 755 nm.
